# Crystal structure, Hirshfeld surface analysis and frontier mol­ecular orbital analysis of (*E*)-4-bromo-*N*′-(2,3-di­chloro­benzyl­idene)benzohydrazide

**DOI:** 10.1107/S2056989019001816

**Published:** 2019-02-05

**Authors:** Palaniyappan Sivajeyanthi, Muthaiah Jeevaraj, Bellarmin Edison, Kasthuri Balasubramani

**Affiliations:** a Department of Chemistry, Government Arts College (Autonomous), Thanthonimalai, Karur- 639 005, Tamil Nadu, India

**Keywords:** crystal structure, Schiff base, hydrogen bonding, Hirshfeld surface analysis

## Abstract

The title Schiff base compound, C_14_H_9_BrCl_2_N_2_O, displays a *trans* or *E* configuration with respect to the C=N bond, with a dihedral angle 15.7 (2)° formed between the benzene rings. In the crystal, mol­ecules are linked by N—H⋯O and C—H⋯O hydrogen bonds, forming chains along [001] which enclose *R* ^1^_2_(6) loops. The inter­molecular inter­actions were investigated by Hirshfeld surfaces analysis and two-dimensional fingerprint plots. The DFT-B3LYP/6–311 G++(d,p) method was used to determine the HOMO–LUMO energy levels.

## Abstract 2

The title Schiff base compound, has an *E* configuration with respect to the C=N bond, and the benzene rings are inclined to each other by 15.7 (2)°. In the crystal, mol­ecules are linked by N—H⋯O and C—H⋯O hydrogen bonds, forming chains along [001] which enclose 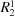
(6) loops.

## Chemical context   

Schiff bases are nitro­gen-containing compounds that were first obtained by the condensation reactions of aromatic amines and aldehydes (Schiff, 1864 ▸). A wide range of these compounds, with the general formula *R*HC=N*R*1 (*R* and *R*1 can be alkyl, aryl, cyclo­alkyl or heterocyclic groups) have been synthesized. They are of great importance in the field of coordination chemistry as they are able to form stable complexes with many metal ions (Souza *et al.*, 1985 ▸). The chemical and biological significance of Schiff bases can be attributed to the presence of a lone electron pair in the *sp* ^2^-hybridized orbital of the nitro­gen atom of the azomethine group (Singh *et al.*, 1975 ▸). These compounds are used in the fields of organic synthesis, chemical catalysis, medicine and pharmacy as well as other new technologies (Tanaka *et al.*, 2010 ▸). Schiff bases are also used as probes in investigating the structure of DNA (Tiwari *et al.*, 2011 ▸) and have gained special attention in pharmacophore research and in the development of several bioactive lead mol­ecules (Muralisankar *et al.*, 2016 ▸). They also exhibit photochromic and thermochromic properties and have been used in information storage, electronic display systems, optical switching devices, and ophthalmic glasses (Amimoto & Kawato, 2005 ▸). Herein, we report on the crystal structure, the Hirshfeld surface analysis and the mol­ecular orbital analysis of the title compound, (*E*)-4-bromo-*N*′-(2,3-di­chloro­benzyl­idene)benzohydrazide.
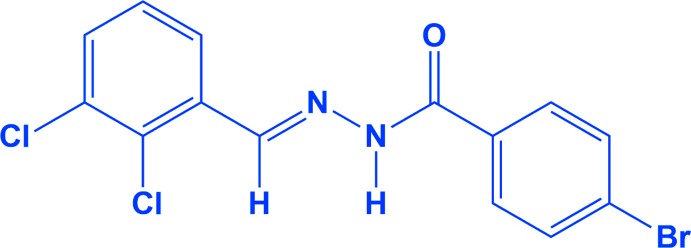


## Structural commentary   

**Figure 1:**
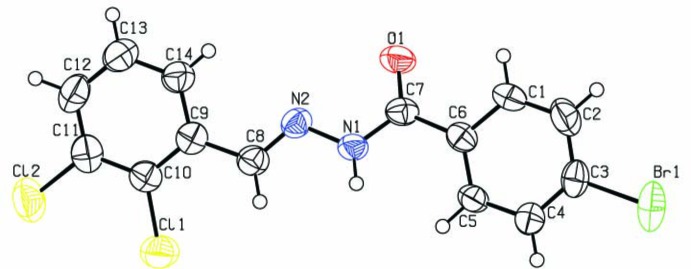
A view of the mol­ecular structure of the title compound, with atom labelling. Displacement ellipsoids are drawn at the 50% probability level.

The mol­ecular structure of the title compound is illustrated in Fig. 1 ▸. The configuration about the C8=N2 bond, which has a bond length of 1.271 (5) Å, is *E*. The benzene rings (C1–C6 and C9–C14) are inclined to each other by 15.7 (2)°. The bond lengths and angles and the overall conformation of the mol­ecule are close to those reported for a very similar compound, (*E*)-4-bromo-*N*′-(2-chloro­benzyl­idene)benzohydrazide (Shu *et al.*, 2009 ▸).

## Supra­molecular features   

**Figure 2:**
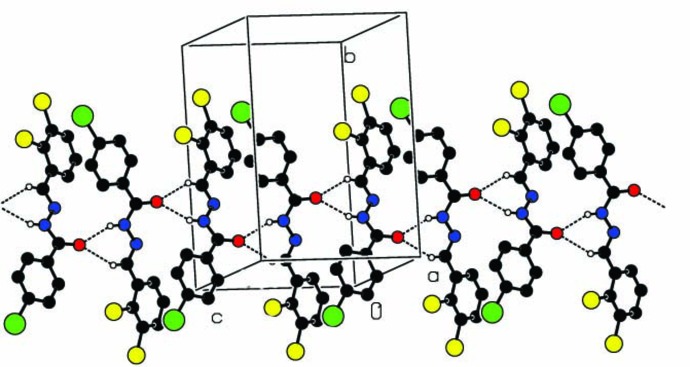
A partial view along the *a* axis of the crystal packing of the title compound. Hydrogen bonds (Table 1 ▸) are shown as dashed lines, and only the H atoms involved in hydrogen bonding have been included.

In the crystal, mol­ecules are linked by N—H⋯O and C—H⋯O hydrogen bonds, forming chains that propagate along the [001] direction and which enclose 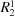
(6) ring motifs (Fig. 2 ▸ and Table 1 ▸). Here the oxygen atom O1 acts as a bifurcated acceptor. There are no other significant inter­molecular inter­actions present (see Table 2 ▸ in *Hirshfeld surface analysis*).

## Hirshfeld surface analysis   

**Figure 3:**
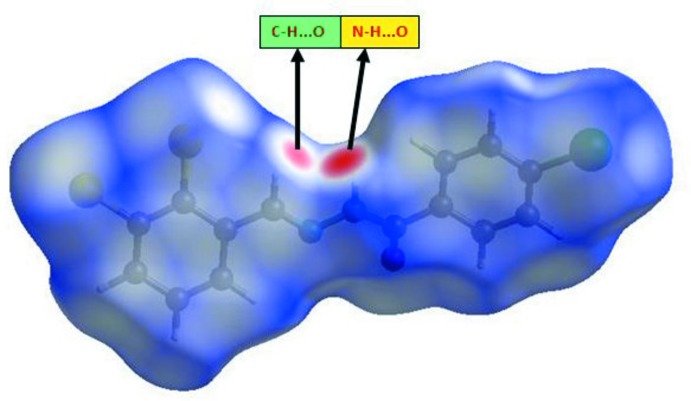
Hirshfeld surface mapped over *d*_norm_ for the title compound. **[add range of dnorm to legend]**

*Crystal Explorer* (Wolff *et al.*, 2012 ▸) was used to generate the Hirshfeld surface and two-dimensional fingerprint plots (Rohl *et al.*, 2008 ▸). The three-dimensional *d*_norm_ surface is a useful tool for analysing and visualizing the inter­molecular inter­actions, which are given in Table 2 ▸. The *d*_norm_ values are negative or positive depending on whether the inter­molecular contact is shorter or longer than the sum of the van der Waals radii (Spackman & Jayatilaka, 2009 ▸; McKinnon *et al.*, 2007 ▸). The total *d*_norm_ surface of the title compound is shown in Fig. 3 ▸. The red spots correspond to the N—H⋯O and C—H⋯O inter­actions, the most significant inter­actions in the crystal (Tables 1 ▸ and 2 ▸).

**Figure 4:**
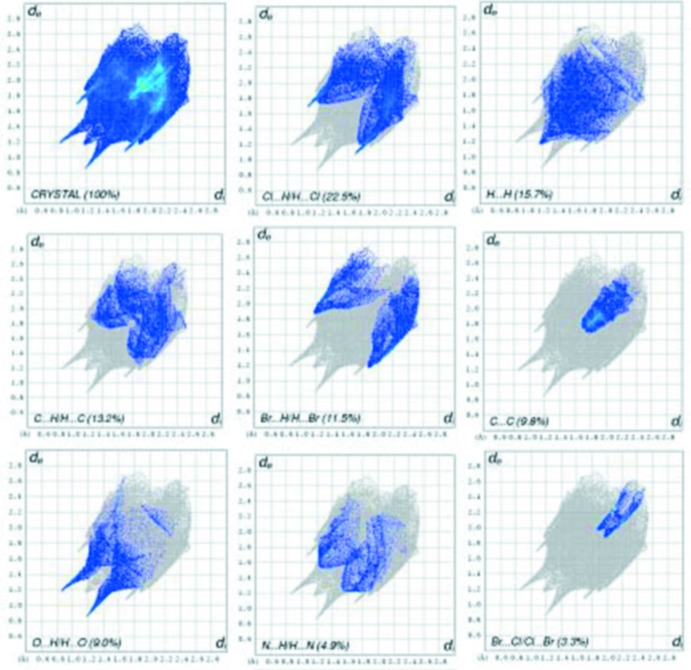
Two-dimensional fingerprint plots of the crystal with the relative contributions of the atom pairs to the Hirshfeld surface.

The two-dimensional fingerprint plots from the Hirshfeld surface analysis are shown in Fig. 4 ▸. They indicate the percentage contributions of the various inter­molecular contacts to the Hirshfeld surface, the most significant are Cl⋯H/H⋯Cl (22.5%), H⋯H (15.7%), C⋯H/H⋯C (13.2%), Br⋯H/H⋯Br (11.5%), C⋯C (9.8%), O⋯H/H⋯O (9.0%), N⋯H/H⋯N (4.9%), and Br⋯Cl/Cl⋯Br (3.3%), as shown in Fig. 4 ▸, *cf* Table 2 ▸.

## Frontier mol­ecular orbital calculations   

**Figure 5:**
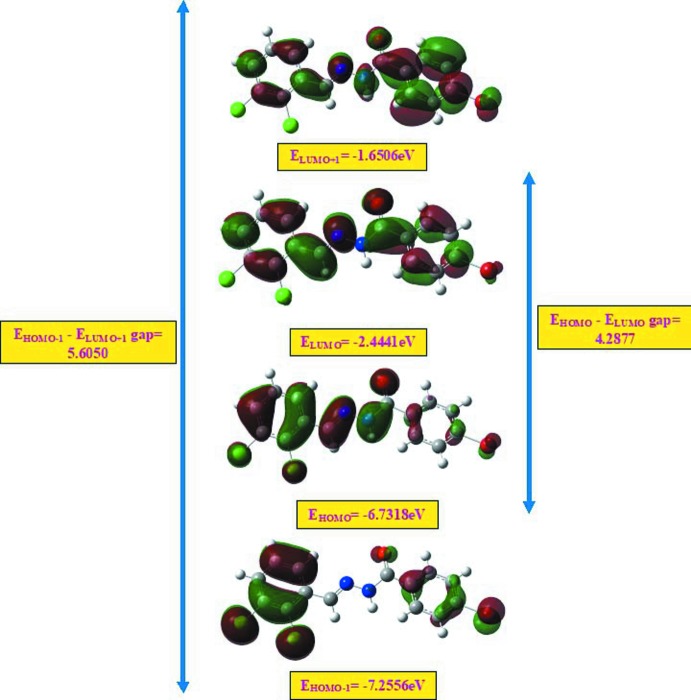
Mol­ecular orbital energy levels of the title compound.

The HOMO (highest occupied mol­ecular orbital) acts as an electron donor and the LUMO (lowest occupied mol­ecular orbital) as an electron acceptor. If the energy gap is small then the mol­ecule is highly polarizable and has high chemical reactivity. The energy levels of the title compound were computed using the DFT-B3LYP/6-311G++(d,p) method (Sivajeyanthi *et al.*, 2017 ▸). The energy gap between HOMO–LUMO orbitals, which determines the chemical stability, chemical hardness, chemical potential, electronegativity and the electrophilicity index are shown in Fig. 5 ▸ and details are given in Table 3 ▸. The frontier mol­ecular orbital LUMO is located over the whole of the mol­ecule. The energy gap of the mol­ecule clearly shows the charge-transfer inter­action involving donor and acceptor groups. The chemical hardness and softness of a mol­ecule is a sign of its chemical stability. From the HOMO–LUMO energy gap, we can see whether or not the mol­ecule is hard or soft. If the energy gap is large, the mol­ecule is hard and if small the mol­ecule is soft. Soft mol­ecules are more polarizable than hard ones because they need less energy for excitation. From the data presented in Table 3 ▸, we conclude that the energy gap is large, hence the title mol­ecule is a hard material and will be difficult to polarize.

## Database survey   

A search of the Cambridge Structural Database (CSD, version 5.39, last update August 2018; Groom *et al.*, 2016 ▸) for 4-bromo-(benzyl­idene)benzohydrazides yielded six structures. They include the following analogues: 2,4-di­hydroxy­benzyl­idene [ATOSEJ (Mohanraj *et al.*, 2016 ▸) and ATOSEJ01 (Arunagiri *et al.*, 2018 ▸)], 2-nitro­benzyl­idene (EGUSEF; Zhang *et al.*, 2009 ▸), 2-chloro­benzyl­idene (HOTDAW; Shu *et al.*, 2009 ▸), 2-hy­droxy-1-naphthyl­methyl­ene (IFUSEI; Diao *et al.*, 2008 ▸), 2-hy­droxy-5-meth­oxy­benzyl­idene (OBUBUL; Wang *et al.*, 2017 ▸) and 4-hy­droxy-3-meth­oxy­benzyl­idene (YAWXOL; Horkaew *et al.*, 2012 ▸). They all have an *E* configuration about the C=N bond. The N—N bond lengths vary from 1.366 (4) to 1.396 (5) Å while the C=N bond lengths vary from 1.264 (4) to 1.285 (2) Å. The values observed for the title compound, respectively, 1.391 (4) and 1.271 (5) Å, fall within these limits. The dihedral angle between the two benzene rings varies from as little as 4.12 (17)° in EGUSEF to 49.08 (18)° in ATOSEJ01. In the title compound this dihedral angle is 15.7 (2)°, similar to the values observed for HOTDAW, the 2-chloro­benzyl­idene analogue, and for YAWXOL, the 4-hy­droxy-3-meth­oxy­benzyl­idene analogue, for which the dihedral angles are 11.43 (16) and 13.92 (6)°, respectively.

## Synthesis and crystallization   

The title compound was synthesized by the reaction of 1:1 molar ratio mixture of a hot ethano­lic solution (20 ml) of 4-bromo­benzohydrazide (0.213 mg, Aldrich) and 2,3-di­chloro­benzaldehyde (0.175 mg, Aldrich), which was refluxed for 8 h. The solution was then cooled and kept at room temperature. The powder obtained was recrystallized from dimethyl sulfoxide (DMSO). Colourless block-like crystals suitable for the X-ray diffraction analysis were obtained in a few days.

## Refinement   

Crystal data, data collection and structure refinement details are summarized in Table 4 ▸. The hydrogen atoms were positioned geometrically and refined using a riding model: C—H = 0.93 Å, N—H = 0.86 Å, with *U*_iso_(H) = 1.2*U*_eq_(N, C).

## Supplementary Material

Crystal structure: contains datablock(s) global, I, 1. DOI: 10.1107/S2056989019001816/su5466sup1.cif

Structure factors: contains datablock(s) I. DOI: 10.1107/S2056989019001816/su5466Isup2.hkl

Supporting information file. DOI: 10.1107/S2056989019001816/su5466Isup3.cml

CCDC reference: 1587252

Additional supporting information:  crystallographic information; 3D view; checkCIF report

## Figures and Tables

**Table 1:** Hydrogen-bond geometry (Å, °)

*D*—H⋯*A*	*D*—H	H⋯*A*	*D*⋯*A*	*D*—H⋯*A*
N1—H1*N*1⋯O1^i^	0.86	2.20	3.003 (4)	155
C8—H8⋯O1^i^	0.93	2.42	3.234 (5)	146

Symmetry code: (i) 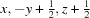.

**Table 2:** Inter­molecular contacts (Å) for the title compound

Atom1⋯Atom2	Length	Length − vdW radii	Symm. op. 2
H1*N*1⋯H5	2.136	−0.264	*x*, *y*, *z*
O1⋯H1*N*1	2.200	−0.520	*x*, 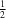 − *y*, *z* − 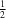
H1*N*1⋯H8	2.242	−0.158	*x*, *y*, *z*
O1⋯H8	2.421	−0.299	*x*, 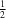 − *y*, *z* − 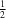
O1⋯H1	2.520	−0.200	*x*, *y*, *z*
N2⋯H14	2.523	−0.227	*x*, *y*, *z*
H1*N*1⋯C5	2.608	−0.292	*x*, *y*, *z*
N1⋯H5	2.652	−0.098	*x*, *y*, *z*
Cl1⋯H8	2.733	−0.217	*x*, *y*, *z*
H1⋯Cl1	2.931	−0.019	*x*, 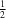 − *y*, *z* − 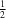
O1⋯N1	3.003 (4)	−0.067	*x*, 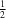 − *y*, *z* − 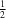
H12⋯Cl2	3.024	0.074	*x*, 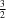 − *y*, *z* − 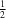
O1⋯C8	3.234 (5)	0.014	*x*, 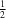 − *y*, *z* − 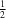
N2⋯C5	3.262 (5)	0.012	*x*, 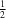 − *y*, *z* − 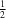
C12⋯Cl2	3.440 (5)	−0.010	*x*, 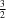 − *y*, *z* − 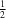
C9⋯C4	3.468 (6)	0.068	*x*, 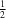 − *y*, *z* − 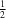
C8⋯C12	3.475 (5)	0.075	−*x*, 1 − *y*, −*z*

**Table 3:** Calculated frontier mol­ecular orbital analysis of the title compound

EHOMO	−6.7318 eV
ELUMO	−2.4441 eV
EHOMO-1	−7.2556 eV
ELUMO+1	−1.6506 eV
EHOMO–ELUMO gap	4.2877 eV
EHOMO−1 ELUMO+1 gap	5.6050 eV
Chemical hardness (η)	2.1438 eV
Chemical potential (μ)	4.5879 eV
Electronegativity (χ)	−4.5879 eV
Electrophilicity index (ω)	4.9092 eV

**Table 4:** Experimental details

Crystal data	
Chemical formula	C_14_H_9_BrCl_2_N_2_O
*M*_r_	372.04
Crystal system, space group	Monoclinic, *P*2_1_/*c*
Temperature (K)	296
*a*, *b*, *c* (Å)	11.1952 (18), 14.055 (2), 9.3050 (12)
β (°)	96.446 (6)
*V* (Å^3^)	1454.8 (4)
*Z*	4
Radiation type	Mo *K*α
μ (mm^−1^)	3.19
Crystal size (mm)	0.30 × 0.20 × 0.20

Data collection	
Diffractometer	Bruker Kappa APEXII CCD
Absorption correction	Multi-scan (*SADABS*; Bruker, 2004 ▸)
*T*_min_, *T*_max_	0.448, 0.568
No. of measured, independent and observed [*I* > 2σ(*I*)] reflections	11392, 3363, 1724
*R*_int_	0.050
(sin θ/λ)_max_ (Å^−1^)	0.666

Refinement	
*R*[*F* ^2^ > 2σ(*F* ^2^)], *wR*(*F* ^2^), *S*	0.051, 0.173, 0.94
No. of reflections	3363
No. of parameters	181
H-atom treatment	H-atom parameters constrained
Δρ_max_, Δρ_min_ (e Å^−3^)	0.70, −0.63

Computer programs: *APEX2*, *SAINT* and *XPREP* (Bruker, 2004 ▸), *SIR92* (Altomare *et al.*, 1993 ▸), *SHELXL2017* (Sheldrick, 2015 ▸) and *PLATON* (Spek, 2009 ▸).

## supplementary crystallographic information

### Crystal data

**Table:**

C_14_H_9_BrCl_2_N_2_O	*F*(000) = 736
*M**_r_* = 372.04	*D*_x_ = 1.699 Mg m^−^^3^
Monoclinic, *P*2_1_/*c*	Mo *K*α radiation, λ = 0.71073 Å
*a* = 11.1952 (18) Å	Cell parameters from 3186 reflections
*b* = 14.055 (2) Å	θ = 4.7–47.5°
*c* = 9.3050 (12) Å	µ = 3.19 mm^−^^1^
β = 96.446 (6)°	*T* = 296 K
*V* = 1454.8 (4) Å^3^	Block, colourless
*Z* = 4	0.30 × 0.20 × 0.20 mm

### Data collection

**Table:**

Bruker Kappa APEXII CCD diffractometer	1724 reflections with *I* > 2σ(*I*)
Radiation source: fine-focus sealed tube	*R*_int_ = 0.050
ω and φ scan	θ_max_ = 28.3°, θ_min_ = 2.6°
Absorption correction: multi-scan (SADABS; Bruker, 2004)	*h* = −14→14
*T*_min_ = 0.448, *T*_max_ = 0.568	*k* = −18→18
11392 measured reflections	*l* = −8→12
3363 independent reflections	

### Refinement

**Table:**

Refinement on *F*^2^	Primary atom site location: structure-invariant direct methods
Least-squares matrix: full	Secondary atom site location: difference Fourier map
*R*[*F*^2^ > 2σ(*F*^2^)] = 0.051	Hydrogen site location: inferred from neighbouring sites
*wR*(*F*^2^) = 0.173	H-atom parameters constrained
*S* = 0.94	*w* = 1/[σ^2^(*F*_o_^2^) + (0.1*P*)^2^] where *P* = (*F*_o_^2^ + 2*F*_c_^2^)/3
3363 reflections	(Δ/σ)_max_ < 0.001
181 parameters	Δρ_max_ = 0.70 e Å^−^^3^
0 restraints	Δρ_min_ = −0.63 e Å^−^^3^

### Special details

**Table:**

Geometry. All esds (except the esd in the dihedral angle between two l.s. planes) are estimated using the full covariance matrix. The cell esds are taken into account individually in the estimation of esds in distances, angles and torsion angles; correlations between esds in cell parameters are only used when they are defined by crystal symmetry. An approximate (isotropic) treatment of cell esds is used for estimating esds involving l.s. planes.
Refinement. Refinement of F^2^ against ALL reflections. The weighted R-factor wR and goodness of fit S are based on F^2^, conventional R-factors R are based on F, with F set to zero for negative F^2^. The threshold expression of F^2^ > 2sigma(F^2^) is used only for calculating R-factors(gt) etc. and is not relevant to the choice of reflections for refinement. R-factors based on F^2^ are statistically about twice as large as those based on F, and R- factors based on ALL data will be even larger.

### Fractional atomic coordinates and isotropic or equivalent isotropic displacement parameters (Å^2^)

**Table:**

	*x*	*y*	*z*	*U*_iso_*/*U*_eq_	
Br1	0.45376 (6)	−0.17154 (4)	0.33892 (7)	0.0891 (3)	
Cl1	0.24263 (13)	0.60728 (9)	0.12531 (12)	0.0749 (4)	
Cl2	0.11288 (14)	0.77291 (9)	−0.05544 (15)	0.0843 (5)	
O1	0.2713 (3)	0.1659 (2)	−0.1706 (3)	0.0628 (9)	
N1	0.2462 (3)	0.2532 (2)	0.0288 (3)	0.0456 (9)	
H1N1	0.253851	0.257938	0.121571	0.055*	
N2	0.1988 (3)	0.3275 (2)	−0.0585 (4)	0.0478 (9)	
C6	0.3268 (4)	0.0931 (3)	0.0576 (4)	0.0439 (10)	
C9	0.1358 (4)	0.4878 (3)	−0.0799 (4)	0.0470 (10)	
C7	0.2801 (4)	0.1731 (3)	−0.0370 (4)	0.0452 (10)	
C5	0.3717 (4)	0.1033 (3)	0.2017 (4)	0.0473 (10)	
H5	0.375456	0.163419	0.243713	0.057*	
C10	0.1524 (4)	0.5818 (3)	−0.0321 (4)	0.0483 (10)	
C3	0.4067 (4)	−0.0633 (3)	0.2236 (5)	0.0527 (11)	
C4	0.4112 (4)	0.0249 (3)	0.2838 (5)	0.0539 (11)	
H4	0.440892	0.032600	0.380460	0.065*	
C8	0.1896 (4)	0.4073 (3)	0.0037 (4)	0.0496 (11)	
H8	0.216763	0.414398	0.101258	0.060*	
C11	0.0950 (5)	0.6564 (3)	−0.1127 (5)	0.0569 (12)	
C12	0.0226 (5)	0.6387 (4)	−0.2402 (5)	0.0663 (14)	
H12	−0.015996	0.688339	−0.292702	0.080*	
C14	0.0639 (4)	0.4716 (3)	−0.2103 (5)	0.0555 (12)	
H14	0.053241	0.409731	−0.244882	0.067*	
C1	0.3261 (5)	0.0017 (3)	−0.0023 (5)	0.0636 (13)	
H1	0.299196	−0.006402	−0.099732	0.076*	
C13	0.0084 (5)	0.5456 (4)	−0.2885 (5)	0.0674 (14)	
H13	−0.039161	0.533003	−0.374844	0.081*	
C2	0.3643 (5)	−0.0761 (3)	0.0792 (6)	0.0716 (15)	
H2	0.361649	−0.136545	0.038188	0.086*	

### Atomic displacement parameters (Å^2^)

**Table:**

	*U*^11^	*U*^22^	*U*^33^	*U*^12^	*U*^13^	*U*^23^
Br1	0.0922 (5)	0.0604 (4)	0.1158 (6)	0.0313 (3)	0.0164 (4)	0.0248 (3)
Cl1	0.1014 (11)	0.0608 (7)	0.0589 (8)	−0.0126 (7)	−0.0069 (7)	0.0007 (6)
Cl2	0.1127 (13)	0.0482 (7)	0.0933 (10)	0.0101 (7)	0.0175 (9)	0.0065 (6)
O1	0.094 (3)	0.0564 (19)	0.0371 (17)	−0.0015 (16)	0.0027 (16)	−0.0063 (13)
N1	0.062 (2)	0.0421 (19)	0.0319 (17)	−0.0021 (17)	0.0032 (16)	0.0005 (14)
N2	0.052 (2)	0.047 (2)	0.0430 (19)	−0.0024 (17)	0.0010 (17)	0.0065 (16)
C6	0.042 (3)	0.042 (2)	0.048 (2)	−0.0064 (19)	0.0082 (19)	−0.0053 (18)
C9	0.044 (3)	0.052 (2)	0.047 (2)	−0.001 (2)	0.012 (2)	0.0049 (19)
C7	0.049 (3)	0.046 (2)	0.041 (2)	−0.0128 (19)	0.004 (2)	−0.0007 (19)
C5	0.060 (3)	0.040 (2)	0.041 (2)	−0.003 (2)	0.005 (2)	−0.0070 (17)
C10	0.050 (3)	0.052 (2)	0.044 (2)	−0.003 (2)	0.013 (2)	0.0009 (18)
C3	0.041 (3)	0.052 (2)	0.066 (3)	0.013 (2)	0.013 (2)	0.008 (2)
C4	0.061 (3)	0.050 (2)	0.050 (2)	0.009 (2)	0.004 (2)	0.002 (2)
C8	0.059 (3)	0.047 (2)	0.043 (2)	−0.004 (2)	0.007 (2)	0.0023 (19)
C11	0.062 (3)	0.053 (3)	0.060 (3)	0.003 (2)	0.025 (3)	0.010 (2)
C12	0.068 (4)	0.069 (3)	0.062 (3)	0.017 (3)	0.005 (3)	0.019 (3)
C14	0.058 (3)	0.061 (3)	0.047 (3)	0.006 (2)	0.004 (2)	−0.002 (2)
C1	0.085 (4)	0.051 (3)	0.051 (3)	0.000 (3)	−0.008 (2)	−0.018 (2)
C13	0.069 (4)	0.078 (4)	0.053 (3)	0.012 (3)	−0.004 (3)	0.002 (3)
C2	0.076 (4)	0.043 (3)	0.093 (4)	0.006 (2)	−0.004 (3)	−0.017 (3)

### Geometric parameters (Å, º)

**Table:**

Br1—C3	1.901 (4)	C5—H5	0.9300
Cl1—C10	1.721 (4)	C10—C11	1.402 (6)
Cl2—C11	1.727 (5)	C3—C4	1.359 (6)
O1—C7	1.240 (5)	C3—C2	1.385 (6)
N1—C7	1.356 (5)	C4—H4	0.9300
N1—N2	1.391 (4)	C8—H8	0.9300
N1—H1N1	0.8600	C11—C12	1.383 (7)
N2—C8	1.271 (5)	C12—C13	1.386 (7)
C6—C5	1.385 (5)	C12—H12	0.9300
C6—C1	1.400 (5)	C14—C13	1.377 (6)
C6—C7	1.486 (6)	C14—H14	0.9300
C9—C14	1.398 (6)	C1—C2	1.372 (7)
C9—C10	1.400 (6)	C1—H1	0.9300
C9—C8	1.464 (6)	C13—H13	0.9300
C5—C4	1.385 (6)	C2—H2	0.9300
			
C7—N1—N2	117.8 (3)	C3—C4—H4	119.9
C7—N1—H1N1	121.1	C5—C4—H4	119.9
N2—N1—H1N1	121.1	N2—C8—C9	119.3 (4)
C8—N2—N1	116.2 (3)	N2—C8—H8	120.3
C5—C6—C1	117.7 (4)	C9—C8—H8	120.3
C5—C6—C7	124.1 (4)	C12—C11—C10	120.9 (4)
C1—C6—C7	118.2 (4)	C12—C11—Cl2	118.2 (4)
C14—C9—C10	118.2 (4)	C10—C11—Cl2	121.0 (4)
C14—C9—C8	119.9 (4)	C11—C12—C13	118.9 (4)
C10—C9—C8	121.9 (4)	C11—C12—H12	120.6
O1—C7—N1	121.7 (4)	C13—C12—H12	120.6
O1—C7—C6	121.0 (4)	C13—C14—C9	121.2 (4)
N1—C7—C6	117.3 (3)	C13—C14—H14	119.4
C4—C5—C6	120.7 (4)	C9—C14—H14	119.4
C4—C5—H5	119.7	C2—C1—C6	121.6 (4)
C6—C5—H5	119.7	C2—C1—H1	119.2
C9—C10—C11	120.0 (4)	C6—C1—H1	119.2
C9—C10—Cl1	120.7 (3)	C14—C13—C12	120.9 (5)
C11—C10—Cl1	119.3 (3)	C14—C13—H13	119.6
C4—C3—C2	120.7 (4)	C12—C13—H13	119.6
C4—C3—Br1	120.1 (3)	C1—C2—C3	118.9 (4)
C2—C3—Br1	119.1 (3)	C1—C2—H2	120.5
C3—C4—C5	120.3 (4)	C3—C2—H2	120.5
			
C7—N1—N2—C8	−166.9 (4)	C14—C9—C8—N2	19.2 (6)
N2—N1—C7—O1	1.1 (6)	C10—C9—C8—N2	−162.0 (4)
N2—N1—C7—C6	−178.0 (4)	C9—C10—C11—C12	−0.6 (7)
C5—C6—C7—O1	160.5 (4)	Cl1—C10—C11—C12	179.5 (4)
C1—C6—C7—O1	−19.2 (6)	C9—C10—C11—Cl2	179.5 (3)
C5—C6—C7—N1	−20.4 (6)	Cl1—C10—C11—Cl2	−0.4 (5)
C1—C6—C7—N1	160.0 (4)	C10—C11—C12—C13	−0.7 (7)
C1—C6—C5—C4	−1.8 (6)	Cl2—C11—C12—C13	179.2 (4)
C7—C6—C5—C4	178.5 (4)	C10—C9—C14—C13	−1.4 (7)
C14—C9—C10—C11	1.6 (6)	C8—C9—C14—C13	177.5 (4)
C8—C9—C10—C11	−177.3 (4)	C5—C6—C1—C2	2.4 (7)
C14—C9—C10—Cl1	−178.5 (3)	C7—C6—C1—C2	−177.9 (4)
C8—C9—C10—Cl1	2.6 (6)	C9—C14—C13—C12	0.1 (7)
C2—C3—C4—C5	0.9 (7)	C11—C12—C13—C14	0.9 (8)
Br1—C3—C4—C5	−177.0 (3)	C6—C1—C2—C3	−1.4 (8)
C6—C5—C4—C3	0.3 (7)	C4—C3—C2—C1	−0.3 (7)
N1—N2—C8—C9	−177.5 (3)	Br1—C3—C2—C1	177.6 (4)

### Hydrogen-bond geometry (Å, º)

**Table:**

*D*—H···*A*	*D*—H	H···*A*	*D*···*A*	*D*—H···*A*
N1—H1*N*1···O1^i^	0.86	2.20	3.003 (4)	155
C8—H8···O1^i^	0.93	2.42	3.234 (5)	146

Symmetry code: (i) *x*, −*y*+1/2, *z*+1/2.
